# Synthesis of hierarchical MgO based on a cotton template and its adsorption properties for efficient treatment of oilfield wastewater

**DOI:** 10.1039/d0ra04181e

**Published:** 2020-08-04

**Authors:** Ying Tang, Zhaoyi Li, Zhongying Xu, Jie Zhang, Chengtun Qu, Zhifang Zhang

**Affiliations:** College of Chemistry and Chemical Engineering, Xi'an Shiyou University Xi'an Shaanxi 710065 China tangying78@xsyu.edu.cn; State Key Laboratory of Petroleum Pollution Control, CNPC Research Institute of Safety and Environmental Technology Beijing 102206 China; School of Chemistry and Chemical Engineering, Yulin University Yulin 719000 China

## Abstract

A biomorphic MgO nanomaterial was fabricated *via* a facile and low-cost immersion method using cotton as the template. The obtained materials were characterized *via* X-ray diffraction (XRD), Fourier transform infrared spectroscopy (FTIR), scanning electron microscopy (SEM) and N_2_ adsorption–desorption analysis. The as-prepared MgO retained the structure of cotton, with a porous hierarchical structure and a high specific surface area, which endowed it with great potential due to its excellent adsorption properties for the adsorption of additives in oil field wastewater. It also exhibited the maximum adsorption capacity of 391.36 mg g^−1^ for sulfonated lignite. The adsorption process of sulfonated lignite on biomorphic MgO was systematically investigated and was found to obey the pseudo-second-order rate equation and the Langmuir adsorption model. The negative values of Gibbs free energy change (Δ*G*) showed that the adsorption process was feasible and spontaneous. The endothermic process was depicted with a positive value for Δ*H*.

## Abstract 2

A biomorphic MgO nanomaterial was fabricated *via* a facile and low-cost immersion method using cotton as the template.

## Graphical abstract

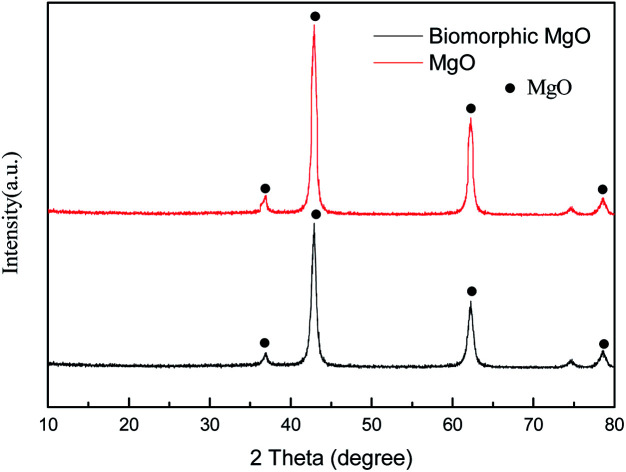

## 1. Introduction

With the rapid development of China's industry, there has been a rapid increase in exploration and exploitation drilling for oil and gas. The amount of drilling wastewater discharge is also growing, resulting in serious environmental problems. Drilling wastewater is industrial wastewater resulting from the process of petroleum drilling, which has a complex composition. It generally contains toxic and harmful substances such as dissolved and dispersed oil sediment, organic and inorganic chemical treatment agents, heavy metal salts, radioactive substances and bacteria, which accumulate in the effluent and in the environment. Furthermore, the impact on flora and fauna is serious due to the direct or chronic toxicity of these compounds and/or their degradation products. Therefore, it is very important to develop technologies to prevent further drilling fluid contamination.

Sulfonated humic acid is a derivative of humic acid and it is also known as sulfonated lignite (SHA), which is formed by the agent (sulfate, sulfite or bisulfite), acid, alkali, formaldehyde, or other inorganic salts under appropriate temperature conditions. Sulfonated lignite is widely used in the drilling mud, which significantly affects the biochemical oxygen demand (BOD), chemical oxygen demand (COD)^1^ and sulfur content of drilling wastewater. Therefore, the study of economical methods for the treatment of sulfonated humic acid in oil field wastewater is of great significance for the protection of water resources and environmental health. To remove these pollutants from drilling wastewater, several conventional methods such as chemical precipitation, oxidation–reduction, electrochemical deposition, filtering, ion exchange, and adsorption have been applied.^2,3^ Among these methods, adsorption, as a more competitive treatment process on an industrial scale, has become an attractive option for industrial wastewater treatment due to its flexibility, simplicity of design and ease of operation.^4^

Numerous advanced porous structure materials have been employed as efficient adsorbents, such as activated carbon.^5,6^ However, its slow sorption kinetics, low regeneration rate and a limited temperature range for optimal operation limit its application in a wide range. Activated carbon is easily affected by the environment during adsorption and leads to adsorbed substances being easily desorbed and released, which again pollute the environment. Chitosan can chemically or physically entrap various metal ions due to the presence of amine and hydroxyl groups that can serve as the chelating and reaction sites.^7^ However, it is difficult to directly apply raw chitosan in the removal of metal ions in wastewater treatment because of its disadvantages such as swelling, solubility in acidic conditions, and unsatisfactory mechanical properties. As a non-toxic, economical, and environmentally friendly material, MgO has already been widely used to treat wastewater^8^ (*e.g.*, for the removal of organic dyes,^9^ toxic fluorides,^10^ heavy metal ions^11,12^ and arsenic).^13^ Despite these successes, the adsorption capacities of the reported MgO materials were not significantly higher than those reported on other adsorbents. Commercial MgO with a small specific surface area and grain size is usually produced by the thermal decomposition of magnesium hydroxide or carbonate, which is disadvantageous for application in heterogeneous systems.^14^

Recent research has shown that hierarchical metal-oxide nanostructured materials as adsorbents can afford remarkable advantages in environmental applications for water purification owing to their large surface areas, high available surface adsorption site density, special functionality, and well-defined morphology. The bio-diversity of natural materials endows us with a wide range of choices for templates with different microstructures. As such, biological templates have attracted significant attention for the fabrication of porous structures. Compared with inorganic templates, biological templates can provide a well-defined morphology in the preparation of micro- and nano-materials. Moreover, the adsorbent synthesized by biological templates can possess a hierarchically porous structure with higher surface area and offers more active sites during the adsorbent process. For example, on employing sorghum straw as a biotemplate, biomorphic porous CaTiO_3_ was prepared by the sol–gel method for adsorbing heavy metal ions from wastewater.^15^ Zhang's group presented the controllable biomimetic fabrication of MgO complex nanostructures with the outstanding ability to adsorb dyes.^16^

In this paper, we developed an effective strategy to synthesize porous hierarchical MgO using cotton as a bio-template. The method involves the impregnation of a matrix with an aqueous precursor solution and the adsorption performance was evaluated by the removal of sulfonated lignite in oilfield wastewater.^17^ Since the effectiveness of adsorption relies on the operational conditions, parameters that may affect the adsorption, including pH and adsorption temperature, were evaluated. Both kinetic and equilibrium isotherm models were applied to establish the rate of adsorption and the adsorption capacity.^18^ The mechanism of scale-up can also serve as the base-line for adsorbent treatment of sulfonated lignite effluents.

## 2. Experimental

### 2.1 Synthesis of the material

Magnesium acetate tetrahydrate (Mg(Ac)_2_·4H_2_O) was purchased from Tianjin Kemiou Chemical Reagent Co. Ltd. Cotton was obtained from Yanggu Jingyangguang Sanitary Material Co. Ltd. Sulfonated lignite was purchased from Tarim, China. All the reagents were of analytical grade and were used as received without further purification. Distilled water was used for all syntheses and treatment processes.

### 2.2 Synthesis of biomorphic MgO

Typically, different dosages of Mg(Ac)_2_·4H_2_O were dissolved in 300 mL distilled water with vigorous stirring to form a homogeneous solution and then 6.7 g cotton was added to the solution. The mixed solution was aged for 24 h at room temperature and dried in a blast drying oven at 70 °C for 12 h. Finally, the sample was calcined at 450, 500 °C and 550 °C for 3 h in an air atmosphere, respectively (with ramping rate 10/min). The template was removed after calcination and biomorphic MgO was thus obtained.

### 2.3 Characterization of materials

The morphology of the prepared material was elucidated using a scanning electron microscope (SEM, JSM-6390A, JEOL, Japan) with an accelerating voltage of 20.0 kV. The phase structures of the calcinated material were analyzed using an X-ray diffraction device (JDX-3530, JEOL, Japan) with an X-ray tube having copper (Cu) as a target and releasing K_α_ radiation when accelerated at 40 mA and 40 kV, at 10–90° with a scanning speed of 2° min^−1^. Surface area and pore property measurements were performed by N_2_ physisorption using a Micromeritics ASAP 2010 instrument at 77 K. The surface areas were calculated using the BET equation in the pressure of range *P*/*P*_0_ = 0.02–0.2, and the pore size distribution was calculated using the Barrett–Joyner–Halenda (BJH) method. The infrared spectra of the samples were measured in the form of KBr (ratio 1 : 100) powder pellets on a Nicolet 5700 Fourier transform infrared (FT-IR) spectrophotometer (Thermo Electron Co., USA) under ambient conditions.

### 2.4 Adsorption experiments

In a typical adsorption experiment procedure, a certain amount of adsorbent was added to 50 mL sulfonated lignite solutions with the initial concentration of 100 mg L^−1^. The mixture was stirred at room temperature (303 K) for 2 h to ensure that adsorption equilibrium was obtained. After that, the sample solutions were filtered, the residual concentrations of sulfonated lignite were measured by a UV-vis spectrophotometer at a maximum adsorption wavelength of 300 nm. The effects of various parameters, such as the solution pH and the dosage on the adsorption capacity in the solution, were studied. The pH of the solution was adjusted with 0.1 mol L^−1^ HCl or 0.1 mol L^−1^ NaOH. For accurate adsorption results, experiments were repeated three times so the average values were represented. The amount of sulfonated lignite adsorbed was calculated based on the formula as given below:1
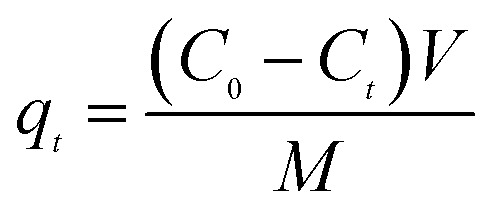
2
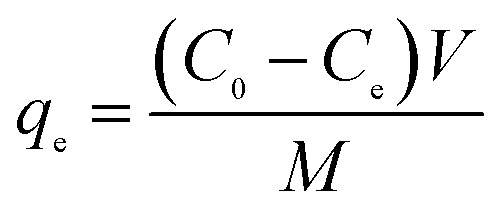
where *C*_0_ and *C*_e_ (mg L^−1^) are the initial and equilibrium sulfonated lignite concentrations respectively; *V* is the volume of solution (mL), and *M* is the mass of adsorbent (g).

## 3. Results and discussion

### 3.1 Structural characterization

#### 3.1.1 XRD analysis

**Fig. 1:**
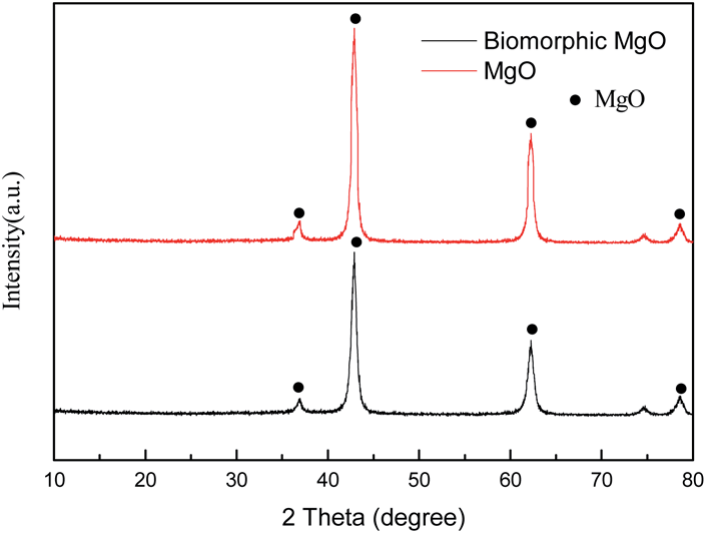
XRD pattern of biomorphic MgO.

The results of the XRD study on the characteristic pattern of MgO calcined directly from the precursor and impregnated cotton are presented in Fig. 1. After dipping degreased cotton in the Mg(CH_3_COOH)_2_·4H_2_O solution, the diffraction peak of the calcined sample was similar to that of commercial MgO with the characteristic diffraction at the (111), (200), (220), and (222) planes of MgO (JCPDS no. 45-0946), indicating that pure MgO can be obtained *via* the thermal decomposition of the Mg(CH_3_COOH)_2_·4H_2_O precursor at 500 °C. Moreover, the relatively broad diffraction peaks revealed that the MgO particles were small crystallites and the grain size was determined to be 11.7 nm by the Scherrer equation (*D* = *Kλ*/*B* cos *θ*). The intensity of the peaks revealed that both samples were crystalline, while the peaks of the biotemplated MgO showed some broadness as compared to commercial MgO, signifying a smaller crystalline size and good dispersion.

#### 3.1.2 Specific surface area and porosity analysis

**Fig. 2:**
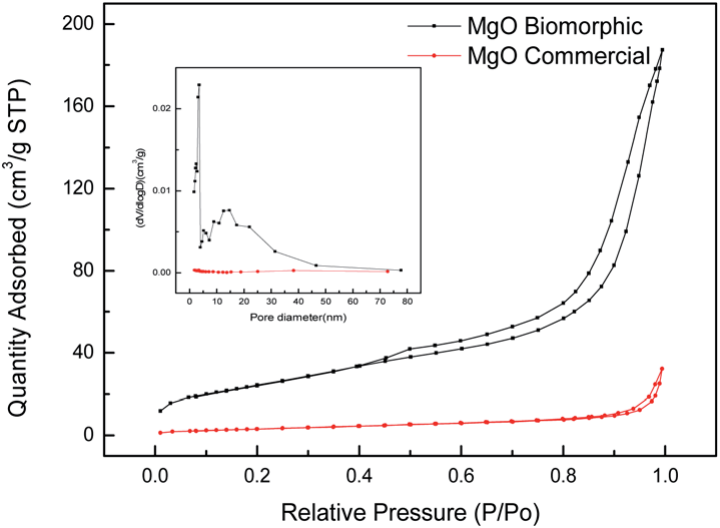
Nitrogen adsorption and desorption isotherms and the corresponding BJH pore size distribution curves of the samples.

To analyze the porous structure of the as-obtained MgO, N_2_ adsorption–desorption measurements were carried out on the sample derived from the cotton template impregnated in 0.01 mol L^−1^ Mg(Ac)_2_·4H_2_O and calcined at 500 °C. The N_2_ adsorption–desorption isotherms and the corresponding BJH pore size distribution curves of the samples are illustrated in Fig. 2. The N_2_ adsorption/desorption isotherm of the biomorphic MgO presented the typical type IV isotherm with a H3-type hysteresis loop, which indicated the presence of mesopores and the adsorption capacity increased rapidly at higher relative pressure due to the existence of macropores. Combined with the pore size distribution curve of biomorphic MgO, it was found that the pore structure distribution of MgO (Fig. 2) exhibited a large number of mesopores and some macropores, confirming the hierarchical meso–macroporous structure of the obtained biomorphic MgO. However, for commercial MgO a small hysteresis loop and narrow pore size distribution indicated its poor pore properties. The specific surface areas, average pore size and total pore volumes of the samples are listed in Table 1. The BET surface area increased from 12.51 cm^2^ g^−1^ to 90.73 cm^2^ g^−1^ and a significant increase from 0.049 to 0.290 cm^3^ g^−1^ in the mesopore volume was observed when we compared commercial MgO with biomorphic MgO. There was a significant overall increase in the surface and porosity of the biomorphic MgO composite, indicating that the utilization of the bio-template technique resulted in the large surface areas of the biomorphic metal oxides.

**Table 1:** BET surface area, pore volume, and pore diameter of different samples

Samples	BET surface area (cm^2^ g^−1^)	Pore volume (cm^3^ g^−1^)	Pore diameter (nm)
Biomorphic MgO	90.7350	0.289915	12.78073
Commercial MgO	12.5065	0.049665	15.88642

#### 3.1.3 SEM analysis

**Fig. 3:**
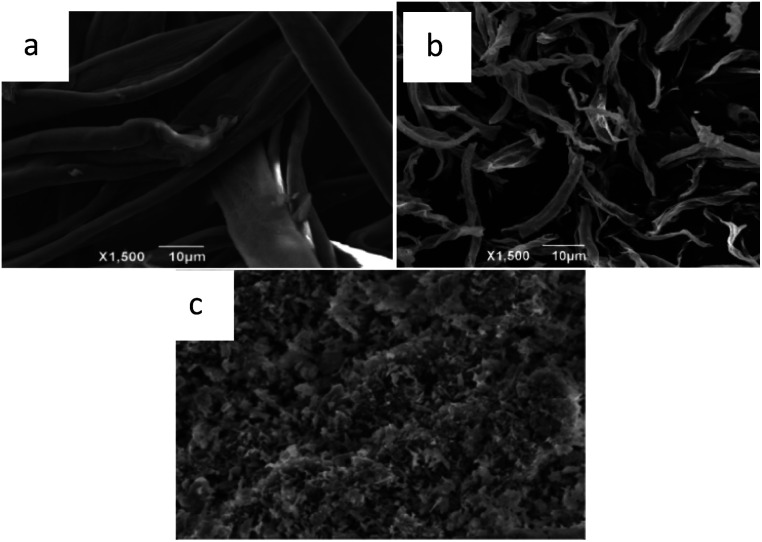
SEM images of (a) cotton, (b) biomorphic MgO, and (c) the commercial MgO product.

The morphologies of cotton fiber, commercial MgO and biomorphic MgO prepared after calcination at 500 °C were investigated by SEM. The cotton had a long tubular microstructure as shown in Fig. 3a with an average diameter of about 10 μm. Fig. 3b illustrates the morphology of the biomorphic MgO. Compared with the diameter of the raw cotton, an apparent volume contraction and collapse were observed, caused by the removal of carbon and volatile organic matter in the calcination process. The biomorphic MgO retained the tubular morphology of cotton and a diameter of about 5 μm by high-temperature calcination. Fig. 3c presents the image of commercial MgO oxides without the template, which revealed an irregular sheet-like structure that was stacked together in a disorderly manner.

### 3.2 Adsorption performance

#### 3.2.1 The effects of different adsorbents

**Fig. 4:**
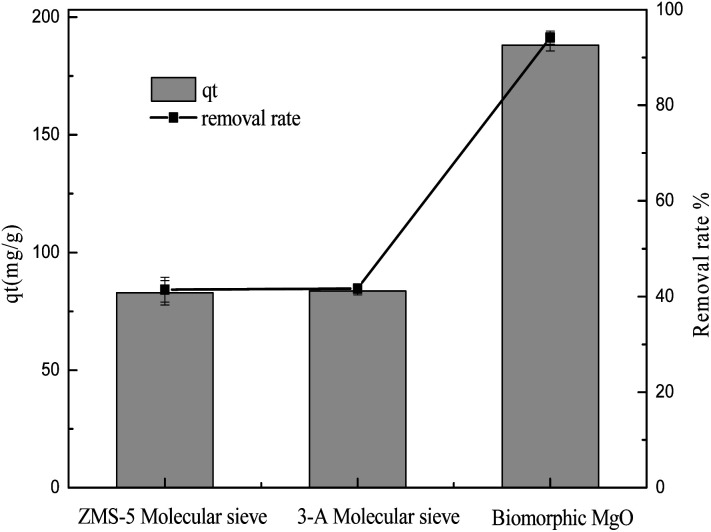
The effects of different adsorbents on the adsorption of sulfonated lignite.

ZSM-5 and 3A molecular sieves, commonly used as adsorbents, were used to compare the adsorption performance with biomorphic MgO in sulfonated lignite solution. As can be seen from Fig. 4, the performances of the different adsorbents on the adsorption capacity of sulfonated lignite were evaluated at pH 6 at 25 °C, and the initial concentration of sulfonated lignite was 100 mg L^−1^. The maximum adsorption capacity was 86.58 and 84.94 mg g^−1^ with the maximum removal rate of 43.29% and 42.27% for ZSM-5 and 3A molecular sieves, respectively. The biomorphic MgO showed better adsorption activity and a 94.33% removal efficiency of sulfonated lignite was obtained, which indicated its good adsorption performance.

#### 3.2.2 The effect of the calcination temperature

**Fig. 5:**
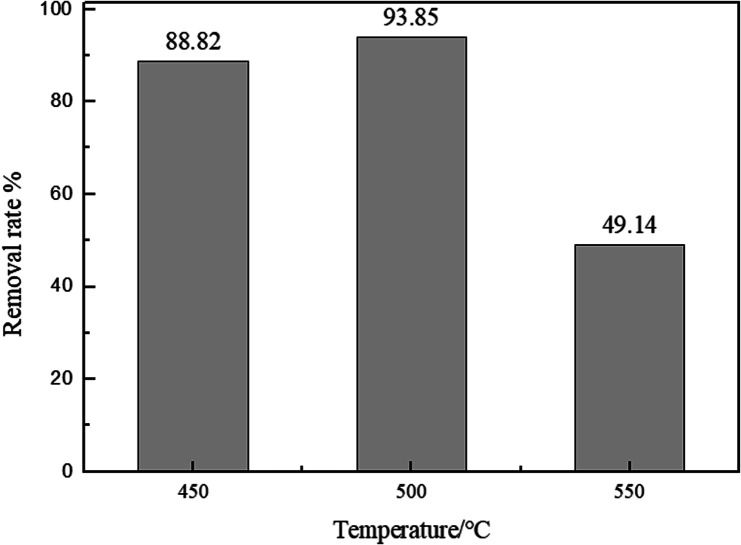
The effect of the calcination temperature on the adsorption of sulfonated lignite over biomorphic MgO.

A significant variable affecting the removal efficiency of adsorption material is the calcination temperature. The adsorption capacities of biomorphic MgO oxides prepared at the calcination temperatures of 450 °C, 500 °C, and 550 °C were compared, using the following reaction conditions: pH = 6, adsorbent dosage of 0.025 g, and initial concentration of 500 mg L^−1^. As shown in Fig. 5, the percentage removal of sulfonated lignite increased from 87.04 to 93.88% when the calcination temperature increased from 450 to 500 °C by the complete conversion of the precursor to MgO(O), while the percentage removal gradually decreased to 44.5% on further increasing the calcination temperature to 550 °C. Compared with the pore properties as shown in Table 1, the biomorphic MgO with large pore size and average volume exhibited the best adsorption efficiency, which further confirmed the strong relationship between the pore size and adsorption property of the adsorbent.

#### 3.2.3 The effect of the precursor concentration

**Fig. 6:**
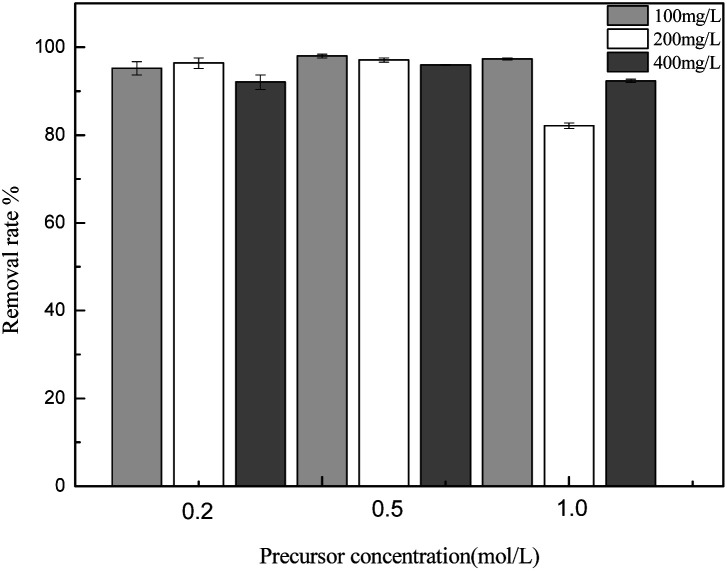
The effect of precursor concentration on the adsorption of sulfonated lignite over biomorphic MgO.

The concentration of the metal salt in the impregnating solution is a vital factor for ascertaining the adsorption rate. The effects of the precursor concentration on the adsorption capacity of sulfonated lignite were investigated at the adsorbent dosage of 0.025 g, 25 °C and initial concentrations of 100, 200, 400 mg L^−1^. The results are presented in Fig. 6. The experimental results indicate that the biomorphic MgO prepared with 0.2 mol L^−1^ magnesium acetate exhibited the highest adsorption capacity with the percentage removal of sulfonated lignite reaching 97.33%. Further increasing the concentration of the precursor resulted in the decrease in the removal rate, due to the increased concentration of metal salt ions causing the enhancement of solution viscosity by incomplete mixing with the template to block the pore structure of the cotton template.

#### 3.2.4 The effect of adsorbent dosage

**Fig. 7:**
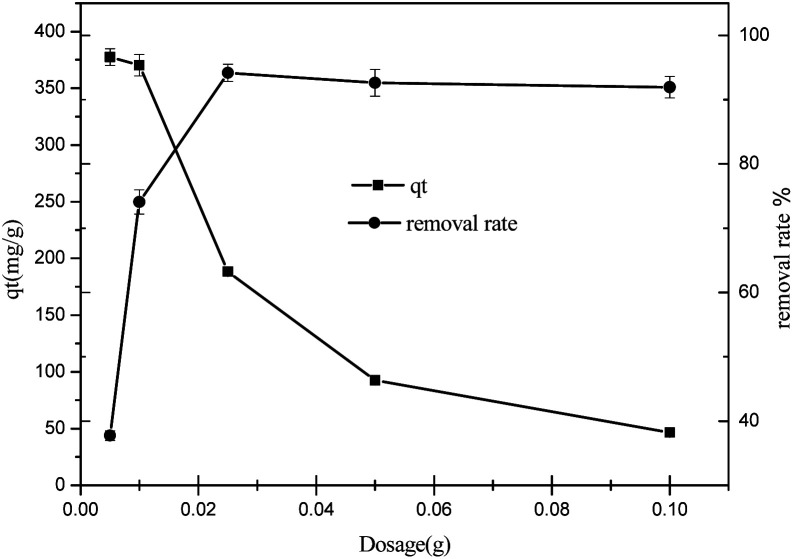
The effect of adsorbent dosage on the adsorption of sulfonated lignite over biomorphic MgO.

The biomorphic MgO concentration is a vital factor for ascertaining the adsorption rate. Fig. 7 shows the adsorption performance of biomorphic MgO investigated by altering the concentration range from 50 mg L^−1^ to 2 g L^−1^ at pH = 6, the temperature of 25 °C, and the initial concentration of sulfonated lignite was 100 mg L^−1^. As seen from the results, the percent adsorption of sulfonated lignite with MgO(O) followed an increasing trend from 37.74 to 94.17%, attributable to the active and available adsorption sites on the porous materials. The adsorption efficiency, as well as the rate of adsorption, was significantly improved when the adsorbent dose reached 250 mg L^−1^ at the sulfonated lignite concentration of 100 mg L^−1^. This can be explained by the positively charged surface of the obtained MgO(O), which facilitates the accessibility of the sulfonated lignite to its large number of active sites and makes favorable conditions for sulfonated lignite adsorption even in the presence of a low adsorbent dosage. In this case, the adsorption on the surface is quickly saturated, showing a high uptake capacity of synthesized MgO(O). At higher particle concentrations, the absorption percent slightly decreased to 91.93%, which is due to the active sites exceeding the demand of the saturated adsorption and resulting in the reduction of the adsorption capacity.^19^ It was concluded that a further increase in the adsorbent dose did not cause any significant increase in the % removal of sulfonated lignite.

#### 3.2.5 The effect of pH

**Fig. 8:**
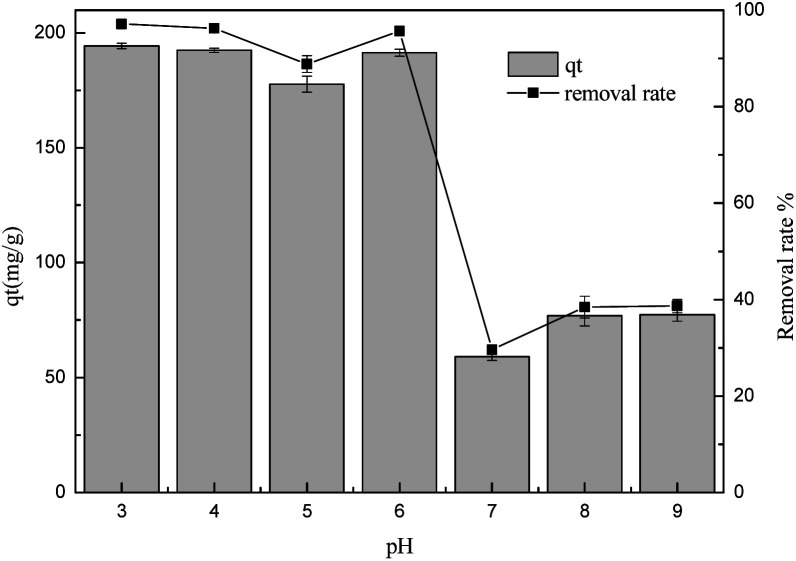
The effect of pH on the adsorption of sulfonated lignite over biomorphic MgO.

The surface charge of the adsorbent is affected by the pH values of the sulfonated lignite solution, so it is very important to investigate the influence of the hydroxyl/hydronium ion concentration on the adsorption process.^20^ The effect of the solution pH on the adsorption capacity of sulfonated lignite over the biomorphic MgO adsorbent was evaluated with the initial pH ranging from 3.0–9.0, the adsorbent dosage of 0.025 g, set temperature of 25 °C and an initial concentration of 100 mg L^−1^. The results are displayed in Fig. 8. The pH of the solution affected the adsorption performance of this adsorbent. The adsorption uptake of biomorphic MgO for sulfonated lignite decreased with the increase in the pH value to the maximum at pH = 3, then it decreased from 194.3 mg g^−1^ to 77.3 mg g^−1^ with an increase in the pH. The isoelectric point (IEP) for magnesium oxide was about 10.5,^21^ which could be because at low pH, the metal oxide surface may attain a positive charge due to the formation ions in a strongly acidic solution. Therefore the electrostatic attraction of the positively-charged metal oxide surfaces and negatively charged adsorbent molecule may lead to the increased uptake efficiency of the sulfonated lignite on the adsorbent.^22^ However, as the pH increases, humic acid may be partially ionized in the solution, resulting in a series of anions, which are difficult to adsorb on the solid surface, thus resulting in the retardation of diffusion and adsorption.^23^

#### 3.2.6 Kinetics of adsorption

To analyze the adsorption rate of sulfonated lignite adsorption onto Mg(O), the kinetic data were modeled using the pseudo-first-order,^24,25^ pseudo-second-order^26,27^ and intra-particle diffusion models.^28^ The linear equations of these three kinetic models are listed below:3
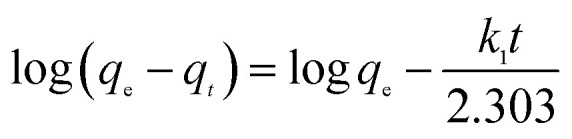
4
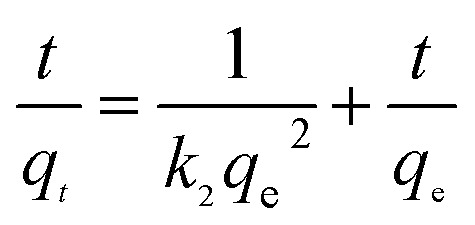
5
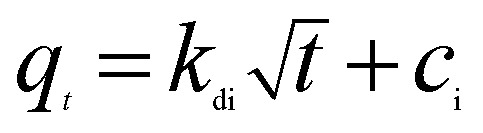
where *q*_*t*_ is the amount of adsorption at time *t*; *k*_1_ (min^−1^) and *k*_2_ (g g^−1^ min) are the rate constants of the pseudo-first-order and pseudo-second-order models, respectively; *k*_di_ and *c*_i_ are the intra-particle diffusion model rate parameter and the intercept of stage i, respectively.

**Fig. 9:**
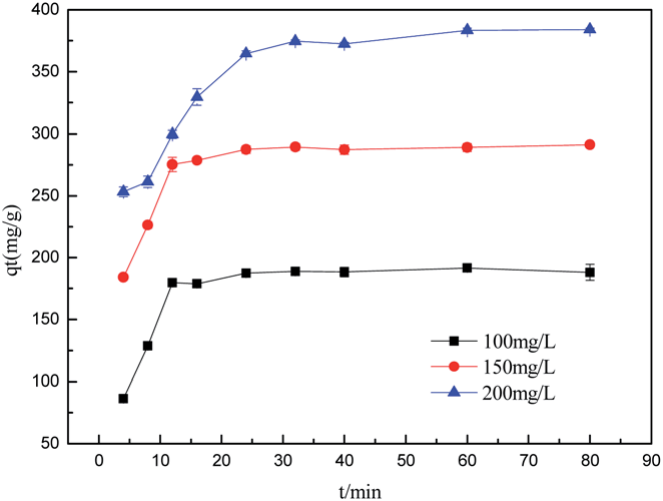
The effect of adsorption time on the adsorption efficiency.

**Fig. 10:**
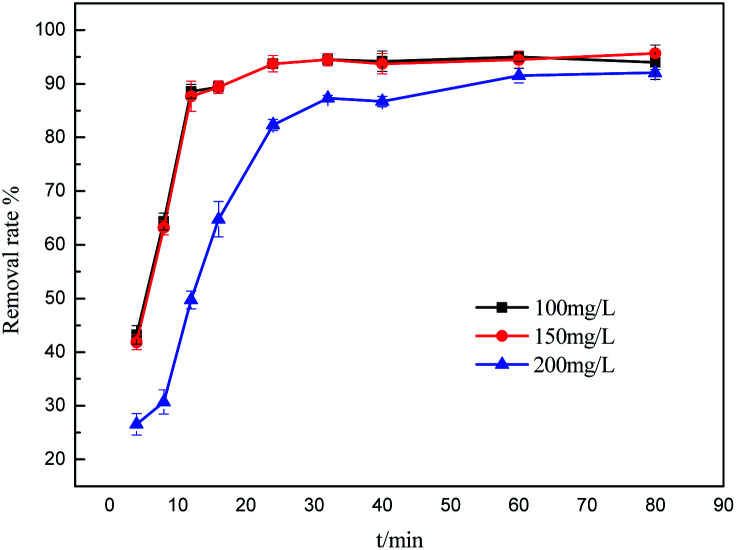
The effect of adsorption time on removal efficiency.

Sulfonated lignite adsorptions on biomorphic MgO at various contact times in solution with initial sulfonated lignite concentrations of 100, 150 and 200 mg L^−1^ are shown in Fig. 9. It can be clearly observed that the adsorption capacity was rapidly enhanced with the contact time increasing from 0 to 16 min. After 40 min of adsorption, the sulfonated lignite adsorption capacity on biomorphic MgO reached 189.63 mg g^−1^, 279.38 mg g^−1^ and 386.34 mg g^−1^, respectively. Further extending the contact time did not significantly improve the sulfonated lignite adsorption capacity, indicating that the hierarchical porous structure can provide more available adsorption sites during the adsorption process (Fig. 10).

**Fig. 11:**
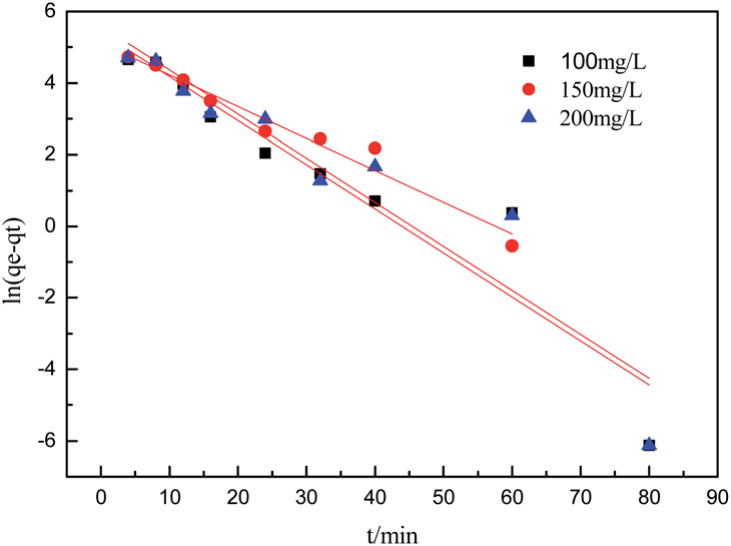
Lagergren first-order kinetic plot for the adsorption of sulfonated lignite over biomorphic MgO.

**Fig. 12:**
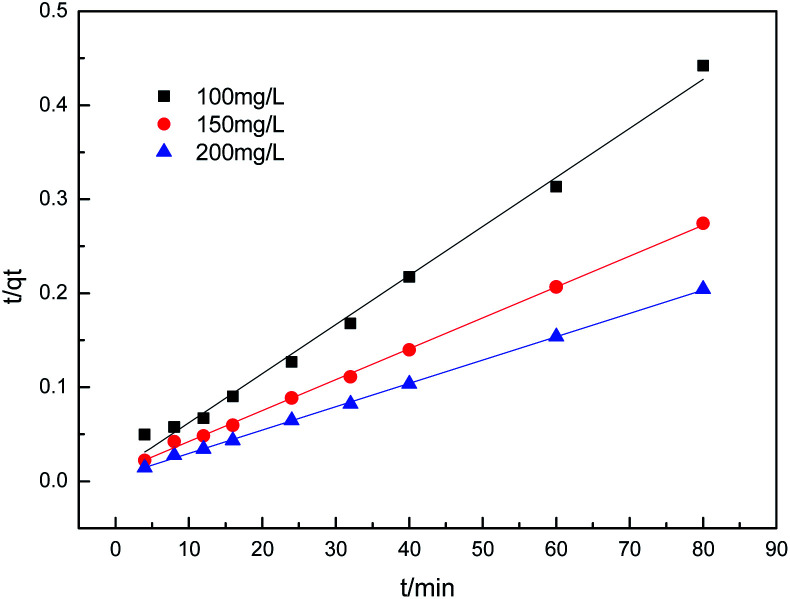
Second-order kinetic plot for the adsorption of sulfonated lignite over biomorphic MgO.

**Fig. 13:**
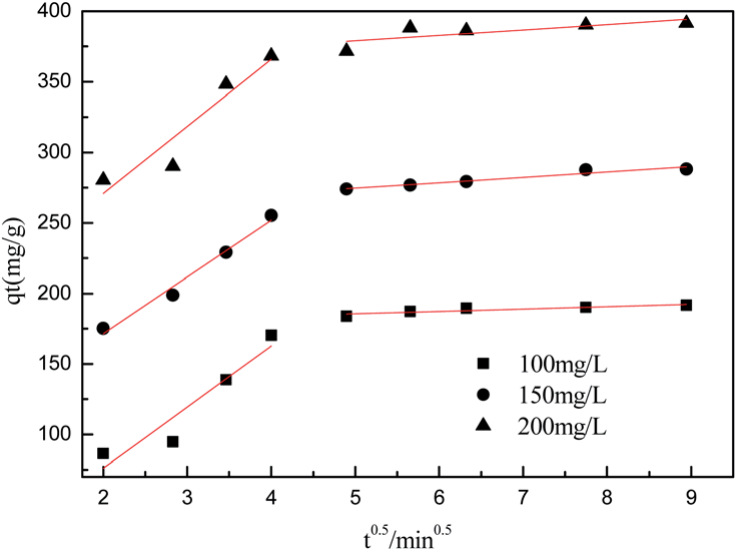
Intraparticle diffusion model for the adsorption of sulfonated lignite over biomorphic MgO.

To further understand the adsorption process, the adsorption kinetics data were fitted to the three models. The results of contact time for the sorption of sulfonated lignite onto the MgO(O) were fitted with the pseudo-first and pseudo-second-order kinetic models and are shown in Fig. 11 and 12. From the slope and intercept of the straight line obtained, the kinetic parameters for the removal of sulfonated lignite were determined, as compiled in Tables 2 and 3. It is apparent that when the pseudo-first-order kinetic model was used, the experimental data (*q*_e_) were much higher than the calculated values, which could be attributed to the low correlation coefficients *R*^2^ in the range 0.8856–0.9605. On the contrary, the pseudo-second-order model showed close agreement between the experimental and calculated adsorption capacity values and gave high *R*^2^ values (*R*^2^ > 0.99), indicating that this kinetic model is suitable for describing the adsorption behavior of sulfonated lignite onto the adsorbents in this study, which showed a monolayer adsorption system. To ensure the adsorption mechanism, the intraparticle diffusion model was investigated based on the resulting sorption data at varying contact times. The intraparticle model kinetics is shown in Fig. 13. Taken as a whole, the linear relations of *q*_*t*_*versus t*^1/2^ are not good. The fact that none of the lines pass through the origin reveals that the intra-particle diffusion is not the rate-controlling step for the whole adsorption process.

**Table 2:** Adsorption kinetic parameters of the pseudo-first-order and pseudo-second-order model for the adsorption of sulfonated lignite onto biomorphic MgO

*C*_0_	*q*_e,exp_	Pseudo-first-order model			Pseudo-second-order model		
		*q*_e,cal_	*K*_1_ min^−1^	*R* ^2^	*q*_e,cal_	*K*_2_ min^−1^	*R* ^2^
100	191.66	225.00	0.123	0.8856	191.57	0.0026	0.9928
150	288.27	167.53	0.089	0.9605	303.95	0.0011	0.9978
200	391.66	269.42	0.123	0.8757	403.22	0.0012	0.9995

**Table 3:** Adsorption kinetic parameters of the intra-particle diffusion model for the adsorption of sulfonated lignite onto biomorphic MgO

Intra-particle diffusion model						
*C*_0_	*K*_d1_	*K*_d2_	*C*_1_	*C*_2_	*R*_1_^2^	*R*_2_^2^
100	43.3658	1.6923	10.7013	177.1835	0.8488	0.7738
200	40.1527	3.8496	97.2479	255.3867	0.9741	0.9286
300	47.4771	3.8303	175.9840	359.9063	0.8500	0.4718

#### 3.2.7 Adsorption isotherms

The analysis of equilibrium data to construct adsorption isotherms is usually important for the design of adsorption systems. Adsorption isotherms express the mathematical relationship between the quantity of adsorbate and the equilibrium concentration of adsorbate remaining in the solution at a constant temperature. Several types of isotherms have been developed for the determination of the equilibrium adsorption capacity of the adsorbent. In this study, the Langmuir (eqn (6)), Freundlich (eqn (7)) and Dubunin–Radushkevich (eqn (8)) models were used to analyze equilibrium adsorption data. The linear forms of these models are expressed as follows:^29,30^6
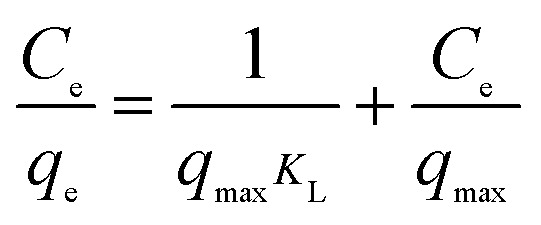
7
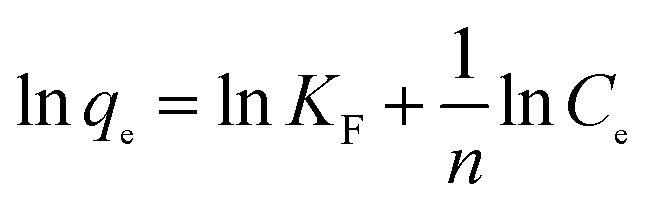

8 D–R:ln *q*_e_ = ln *q*_max_ − *βε*^2^

where *q*_max_ (mg g^−1^) is the theoretical maximum adsorption capacity, *K*_L_ is the Langmuir constant related to the energy of adsorption, *K*_F_ is the Freundlich constant related to adsorption capacity, 1/*n* is the empirical parameter related to adsorption intensity. *β* is a constant associated with the adsorption energy, and *ε* is the Polanyi potential, which is equal to *RT* ln(1 + 1/*C*_e_).

**Fig. 14:**
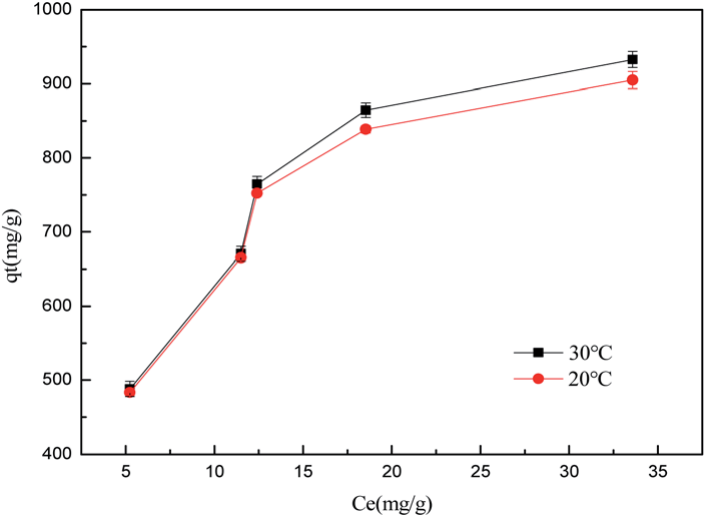
The effect of adsorption temperature on sulfonated lignite removal at different temperatures.

To evaluate the adsorption capacities of sulfonated lignite onto biomorphic MgO, we described the adsorbent–adsorbate interactions and adsorption mechanism. The adsorption isotherms at different temperatures while varying the initial concentrations from 200–500 mg L^−1^ are depicted in Fig. 14. It can be seen that increasing the experimental temperature led to an increase in the adsorption capacity. The maximum adsorption capacity increased from 912.10 to 935.98 mg g^−1^ with the temperature increase from 25 °C to 35 °C at the initial concentration of 500 mg g^−1^. The results indicate that the adsorption reaction of sulfonated lignite adsorbed by biomorphic MgO is an endothermic process. This indicated that high temperature favored the adsorption of sulfonated lignite onto the active sites of Mg(O).

**Fig. 15:**
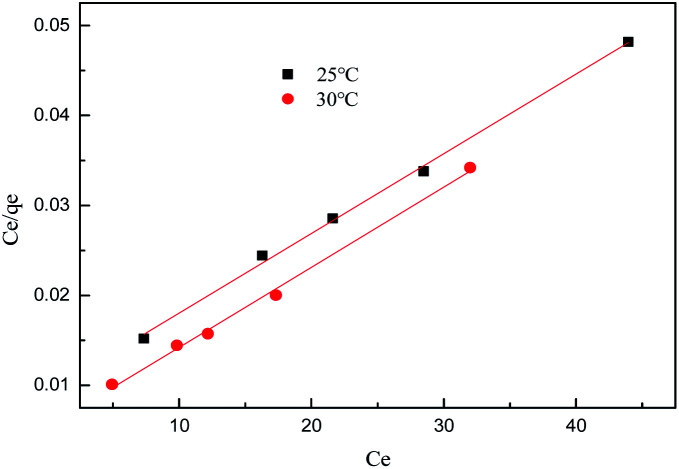
Langmuir adsorption isotherm for sulfonated lignite onto biomorphic MgO at different temperatures.

**Fig. 16:**
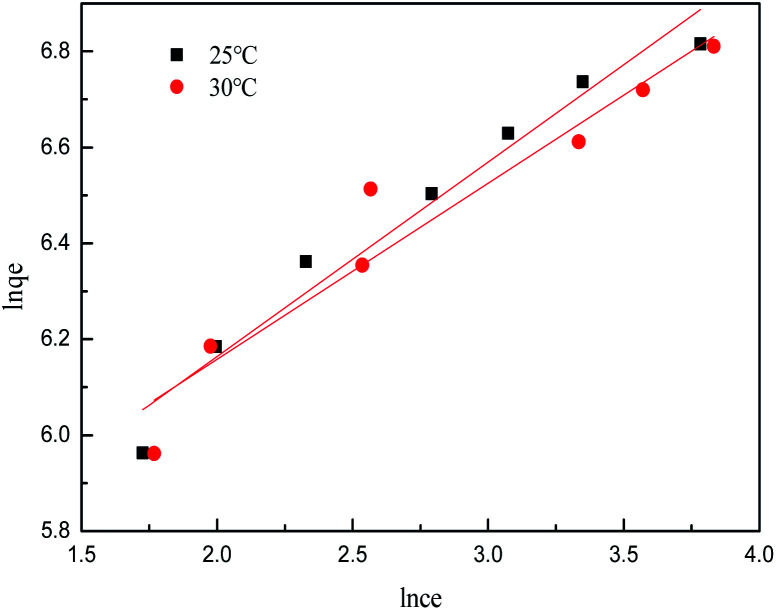
Freundlich adsorption isotherm for sulfonated lignite onto biomorphic MgO at different temperatures.

**Fig. 17:**
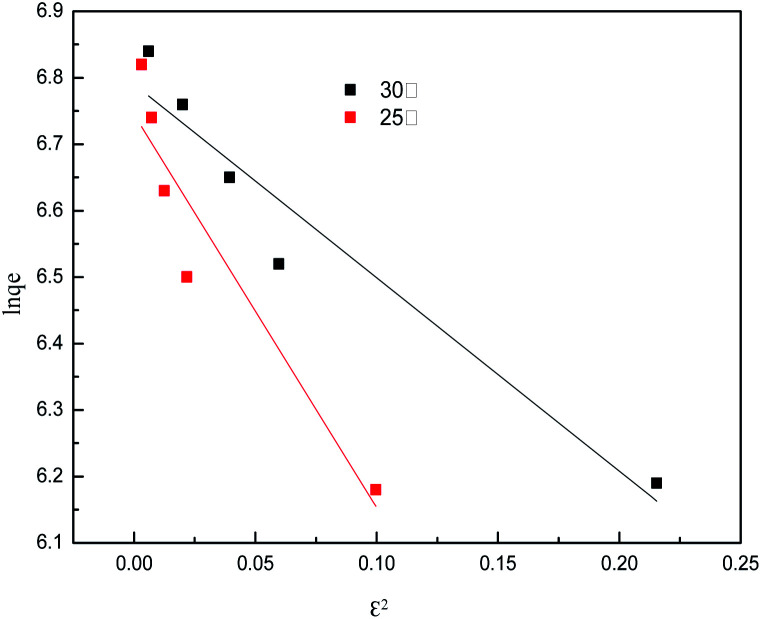
D–R adsorption isotherm for sulfonated lignite onto biomorphic MgO at different temperatures.

The isotherm constants of the Langmuir isotherm, Freundlich isotherm and D–R adsorption isotherm presented in Table 4 were calculated from the slope and intercept of linear plots obtained as shown in Fig. 15–17, respectively. It was concluded from the results that the Langmuir adsorption isotherm (*R*^2^ = 0.9969, 0.9955) was more suitable than the Freundlich adsorption isotherm (*R*^2^ = 0.9579, 0.9188) and the D–R adsorption isotherm (*R*^2^ = 0.8626, 0.9178) in terms of sulfonated lignite adsorption onto the adsorbent, which indicated the monolayer adsorption of the adsorbate with energetically equal sites of well-defined adsorbent with no interaction between the adsorbate molecules.^31^ The parameter *R*_L_ indicates the nature of the shape of the isotherm: *R*_L_ > 1 unfavorable adsorption, 0 < *R*_L_ < 1 favorable adsorption, *R*_L_ = 0 irreversible adsorption, *R*_L_ = 1 linear adsorption. The value of *R*_L_ in the present investigation was 0 < *R*_L_ < 1; therefore, the adsorption process was very favorable and the adsorbent employed exhibited good potential for the removal of sulfonated lignite from aqueous solution.^32^ Thus the Langmuir model is the most applicable for describing sulfonated lignite adsorption onto the biomorphic MgO adsorbent.

**Table 4:** Adsorption isotherm parameters of the Langmuir, Freundlich and D–R models for the adsorption of sulfonated lignite onto biomorphic MgO

*T* (°C)	Langmuir isotherm model			Freundlich isotherm model			D–R isotherm model		
	*q*_max_ (mg g^−1^)	*K*_L_ (L mg^−1^)	*R* ^2^	*K*_F_ (mg g^−1^) (L mg^−1^)^1/*n*^	*n*	*R* ^2^	*q*_max_ (mg g^−1^)	*β*	*R* ^2^
25	1127.75	0.0969	0.9969	238.99	2.732	0.9752	849.62	5.9196	0.8626
30	1121.92	0.168	0.9955	298.22	2.831	0.8966	889.18	2.9133	0.9178

#### 3.2.8 Adsorption thermodynamics

During any adsorption process, the changes in energy and entropy should be considered to determine what process will take place spontaneously and whether the process is spontaneous or not. The values of thermodynamic parameters have great meaning for practical application for adsorption processes.^33^ Experiments were carried out using 500 mg g^−1^ of sulfonated lignite solution and the adsorbent dosage of 0.025 g at temperatures of 20, 30 and 40 °C, respectively. The thermodynamic parameters Gibb's free energy (Δ*G*°, kJ mol^−1^), enthalpy (Δ*H*°, kJ mol^−1^) and entropy (Δ*S*°, kJ mol^−1^ K^−1^) were calculated using the following equations:^34^9
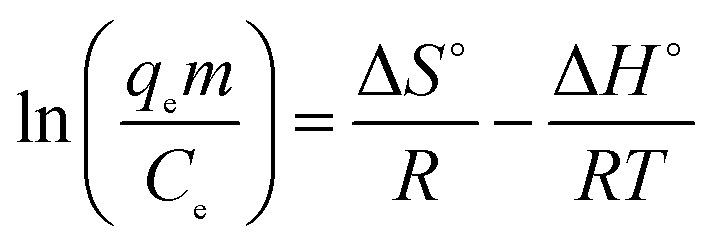

**Fig. 18:**
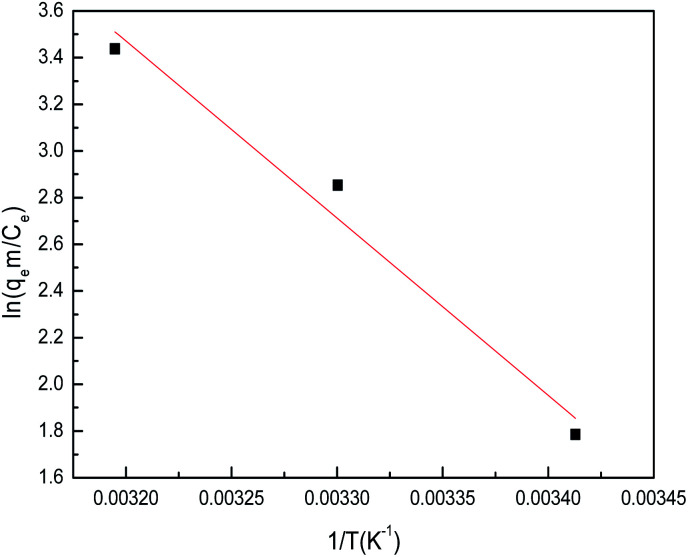
The plots of ln(*q*_e_*m*/*C*_e_) *vs.* 1/*T* for sulfonated lignite onto biomorphic MgO at different temperatures.

10 Δ*G*° = Δ*H*° − *T*Δ*S*°

where *m* is the adsorbent dose (g L^−1^), *C*_e_ is the concentration of metal ions in solution at equilibrium (mg L^−1^), *q*_e_ is the amount of metal ions at equilibrium in unit mass of adsorbent (mg g^−1^), *q*_e_/*C*_e_ is called the adsorption affinity. *R* is the universal gas constant (8.314 J mol^−1^ K^−1^) and *T* is the temperature (K). The values of Δ*G*° and Δ*H*° can be calculated from the slope and intercept of the van't Hoff plot (ln *q*_e_*m*/*C*_e_*vs.* 1/*T*) shown in Fig. 18. The results of Δ*G*°, Δ*S*° and Δ*H*° are shown in Table 5.

**Table 5:** Thermodynamic parameters for the adsorption of sulfonated lignite onto biomorphic MgO

Temperature (K)	Δ*G*° (kJ mol^−1^)	Δ*H*° (kJ mol^−1^)	Δ*S*°(J mol^−1^ K^−1^)
293	−4.35	63.13	230.88
303	−7.18		
313	−8.94		

The results showed that theΔ*G*° values are negative and their absolute values increased with temperature. This suggests that a high temperature is favoured for the adsorption of sulfonated lignite onto biomorphic MgO, which indicates a spontaneous adsorption process. Moreover, the decrease in Δ*G*° as the temperature rises indicates that the adsorption was more favorable at higher temperatures, which is consistent with the results of the more efficient absorption capacity at higher temperatures as shown in Fig. 18. It is well known from the thermodynamics study that if Δ*G*° values fall in the range of −20–0 kJ mol^−1^, then it is the physisorption process, whereas the Δ*G*° values found in this range support physisorption in Mg(O). This observation was further validated by the isotherm studies as described before. The positive values of Δ*H*° suggest the endothermic nature of the adsorption of sulfonated lignite onto biomorphic MgO, indicating the endothermic nature of the adsorption process. The positive values of entropy Δ*S*° show the increasing randomness at the solid/solution interface during the adsorption process. Also, the positive entropy of adsorption reflects the affinity of the adsorbent for the sulfonated lignite.^35^

### 3.3 Adsorption mechanism

**Fig. 19:**
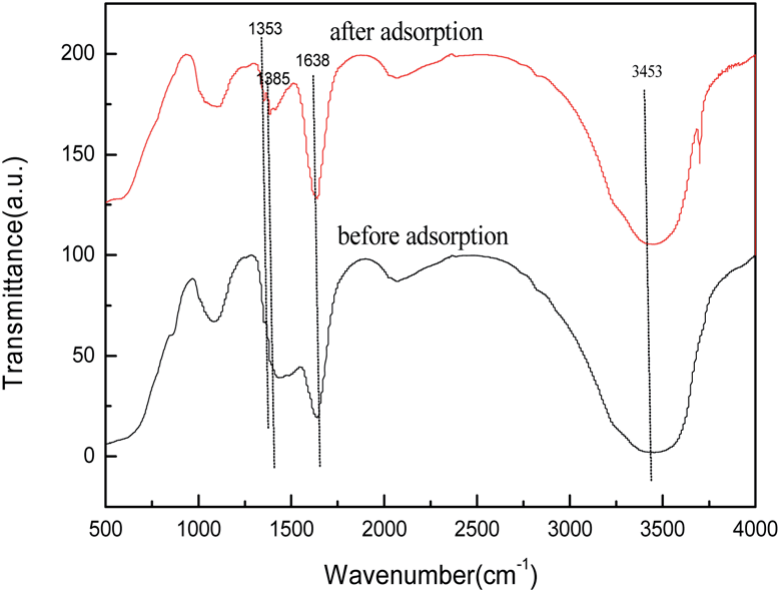
FTIR spectra of biomorphic MgO before and after adsorption.

**Fig. 20:**
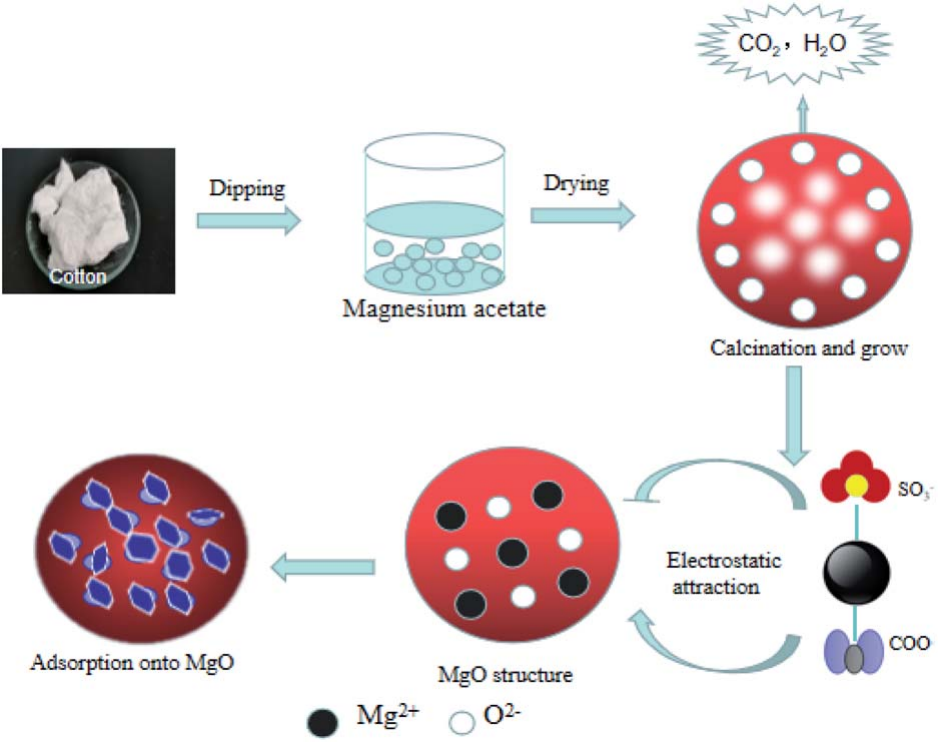
A possible mechanism for the adsorption of sulfonated lignite onto biomorphic MgO.

To better understand the adsorption process, we studied its adsorption mechanism, the FTIR spectra of biomorphic MgO before and after adsorption are shown in Fig. 19. The peak near 1638 cm^−1^ could be attributed to the bending vibration of interlayer water molecules and the peak at 1434 cm^−1^ to the bending vibration of Mg(OH)_2_.^37^ The FTIR spectra of biomorphic MgO after adsorption showed new peaks at 1353 and 1385 cm^−1^, ascribed to the S–O stretching of the sulfonate group in sulfonated lignite and the characteristic symmetrical bending vibration of the carboxylate COO–, suggesting the adsorption of sulfonated lignite onto biomorphic MgO due to the defects of magnesium (V, V^−^, V^2−^) and oxygen holes (F, F^+^, F^2+^) on the surface of nano-magnesium oxide.^36^ In conclusion, the adsorption of sulfonated lignite onto biomorphic MgO could be speculated to occur at the surface of the adsorbent through electrostatic attraction to sulfonated lignite as suggested in Fig. 20.

## 4. Conclusions

In summary, the biomorphic hierarchical MgO was synthesized through an immersion method using cotton as a bio-template. Cotton plays a crucial role in the formation of the biomorphic MgO hierarchical microstructures. The adsorption of sulfonated lignite onto biomorphic MgO depends on the time of contact, the solution pH, and the initial concentrations of sulfonated lignite solution, which followed the pseudo-second-order kinetic model. In addition, under the conditions of 100 mg L^−1^ and 50 mL sulfonated lignite solution with 250 mg adsorption dose, the calcined sample showed better adsorption activity and 94.17% removal efficiency of sulfonated lignite could be obtained. The adsorption of sulfonated lignite onto biomorphic MgO was successfully fit to Langmuir isotherm models over all the concentration ranges studied. The negative values for Δ*G* suggested that the adsorption was spontaneous in nature. The positive values for Δ*H* and Δ*S* indicated an endothermic adsorption process and increased randomness at the surface–solution interface, respectively. This facile preparation method and the improved properties derived from the cotton template are of great significance in the synthesis and application of nanomaterials.

## Conflicts of interest

There are no conflicts to declare.

## Supplementary Material

## References

[cit1] Zhang H., Wang B., Ma H. (2010). Desalination.

[cit2] Hasan S., Abburi K., Ghosh T. K., Viswanath D. S. (2006). Ind. Eng. Chem. Res..

[cit3] Madhava Rao M., Ramana D. K., Seshaiah K., Wang M. C., Chang Chien S. W. (2009). J. Hazard. Mater..

[cit4] Chen L., Bai B. (2013). Ind. Eng. Chem. Res..

[cit5] Chávez-Guerrero L., Rangel-Méndez R., Cullen D. A., Smith D. J., Terrones H., Terrones M. (2008). Water Res..

[cit6] Kazemipour M., Ansari M., Tajrobehkar S., Majdzadeh M., Kermani H. R. (2008). J. Hazard. Mater..

[cit7] Varma A. J., Deshpande S. V., Kennedy J. F. (2004). Carbohydr. Polym..

[cit8] Liu W., Huang F., Wang Y., Zou T., Lin Z. (2011). Environ. Technol..

[cit9] Liu X., Niu C., Zhen X., Wang J., Su X. (2015). J. Mater. Res..

[cit10] Li L., Xu D., Li X., Liu W., Jia Y. (2014). New J. Chem..

[cit11] Cao C., Qu J., Wei F., Liu H., Song W. (2012). ACS Appl. Mater. Interfaces.

[cit12] Chowdhury I. H., Chowdhury A. H., Bose P., Mandal S., Naskar M. K. (2016). RSC Adv..

[cit13] Yang S., Huang P., Peng L., Cao C., Zhu Y., Wei F., Suh Y., Song W. (2016). J. Mater. Chem. A.

[cit14] Sreelatha G., Ageetha V., Parmar J., Padmaja P. (2011). J. Chem. Eng. Data.

[cit15] Vimonses V., Lei S., Jin B., Chow C. W. K., Saint C. (2009). Chem. Eng. J..

[cit16] Zhang D., Wang M., Ren G., Song E. (2013). Mater. Sci. Eng..

[cit17] Li J., Hu M. (2011). Adv. Mater. Res..

[cit18] Bhattacharya A. K., Mandal S. N., Das S. K. (2006). Chem. Eng. J..

[cit19] Gupta V. K., Jain R., Varshney S., Saini V. K. (2007). J. Colloid Interface Sci..

[cit20] Qian Q., Machida M., Aikawa M., Tatsumoto H. (2008). J. Mater. Cycles Waste Manage..

[cit21] Wang B., Xiong X., Ren H., Huang Z. (2017). RSC Adv..

[cit22] Cheng B., Le Y., Cai W., Yu J. (2011). J. Hazard. Mater..

[cit23] Lobrutto R., Jones A., Kazakevich Y. V., McNair H. M. (2001). J. Chromatogr. A.

[cit24] Mittal A., Malviya A., Kaur D., Mittal J., Kurup L. (2007). J. Hazard. Mater..

[cit25] Ho Y. S., Mckay G. (2000). Water Res..

[cit26] Azizian S. (2004). J. Colloid Interface Sci..

[cit27] Lorenc-Grabowska E., Gryglewicz G. (2005). J. Colloid Interface Sci..

[cit28] Guo Y., Zhu Z., Qiu Y., Zhao J. (2013). Chem. Eng. J..

[cit29] Gupta V. K., Pathania D., Sharma S., Singh P. (2013). J. Colloid Interface Sci..

[cit30] Bhattacharya A. K., Mandal S. N., Das S. K. (2006). Chem. Eng. J..

[cit31] Shu J., Wang Z., Huang Y., Huang N., Ren C., Zhang W. (2015). J. Alloys Compd..

[cit32] Feng Y., Gong J., Zeng G., Niu Q., Zhang H., Niu C. (2010). Chem. Eng. J..

[cit33] Nasirimoghaddam S., Zeinali S., Sabbaghi S. (2015). J. Ind. Eng. Chem..

[cit34] Meena A. K., Mishra G. K., Rai P. K., Rajagopal C., Nagar P. N. (2005). J. Hazard. Mater..

[cit35] Jain C. K., Singhal D. C., Sharma M. K. (2004). J. Hazard. Mater..

[cit36] Kim Y. D., Stultz J., Goodman D. W. (2007). J. Phys. Chem..

[cit37] Xiong Y., Wu B., Zhu J., Fan X., Cai P., Wen J., Liu X. (2014). Hydrometallurgy.

